# P2X7 Receptor and Caspase 1 Activation Are Central to Airway Inflammation Observed after Exposure to Tobacco Smoke

**DOI:** 10.1371/journal.pone.0024097

**Published:** 2011-09-06

**Authors:** Suffwan Eltom, Christopher S. Stevenson, Joseph Rastrick, Nicole Dale, Kristof Raemdonck, Sissie Wong, Matthew C. Catley, Maria G. Belvisi, Mark A. Birrell

**Affiliations:** 1 Respiratory Pharmacology, Airway Disease Section, National Heart and Lung Institute, Faculty of Medicine, Imperial College London, London, United Kingdom; 2 Centre for Integrative Mammalian Physiology and Pharmacology, Imperial College London, London, United Kingdom; 3 Union Chimique Belge Pharma Ltd, Union Chimique Belge Celltech, Slough, Berkshire, Belgium; Fundação Oswaldo Cruz, Brazil

## Abstract

Chronic Obstructive Pulmonary Disease (COPD) is a cigarette smoke (CS)-driven inflammatory airway disease with an increasing global prevalence. Currently there is no effective medication to stop the relentless progression of this disease. It has recently been shown that an activator of the P2X7/inflammasome pathway, ATP, and the resultant products (IL-1β/IL-18) are increased in COPD patients. The aim of this study was to determine whether activation of the P2X7/caspase 1 pathway has a functional role in CS-induced airway inflammation. Mice were exposed to CS twice a day to induce COPD-like inflammation and the role of the P2X7 receptor was investigated. We have demonstrated that CS-induced neutrophilia in a pre-clinical model is temporally associated with markers of inflammasome activation, (increased caspase 1 activity and release of IL-1β/IL-18) in the lungs. A selective P2X7 receptor antagonist and mice genetically modified so that the P2X7 receptors were non-functional attenuated caspase 1 activation, IL-1β release and airway neutrophilia. Furthermore, we demonstrated that the role of this pathway was not restricted to early stages of disease development by showing increased caspase 1 activation in lungs from a more chronic exposure to CS and from patients with COPD. This translational data suggests the P2X7/Inflammasome pathway plays an ongoing role in disease pathogenesis. These results advocate the critical role of the P2X7/caspase 1 axis in CS-induced inflammation, highlighting this as a possible therapeutic target in combating COPD.

## Introduction

Chronic Obstructive Pulmonary Disease (COPD) is an inflammatory disease of the airways, associated primarily with cigarette smoke (CS) exposure, and characterised by a progressive and irreversible decline in lung function caused by airflow obstruction, destruction of parenchyma and emphysema [1], [2]. It is one of the few leading global causes of death that is still increasing in prevalence and is predicted to be the third leading cause of mortality by the year 2020 [3]. Studies examining the lungs of COPD patients have demonstrated the infiltration of immune cells including CD8^+^ T-cells, neutrophils and macrophages and as such inflammatory cells have been implicated in the pathogenesis of COPD [4]. This hypothesis, however, is yet to be tested because there are currently no medications that will reduce the airway inflammation. Indeed, even high systemic doses of glucocortoid treatment have limited effects [5]. Therefore there is an urgent need to develop effective anti-inflammatory drugs for patients suffering from COPD. To do this we would argue that it is essential to determine the mechanisms by which exposure to CS drives the airway inflammation.

 Recently there has been growing evidence to implicate the NLRP3 inflammasome and its products in the inflammation observed in COPD patients. The NLRP3 inflammasome is a multi-meric protein complex important in stimulating caspase-1 activation and subsequently the release of the mature form of the inflammatory cytokines IL-1β and IL-18. Elevated IL-1β levels are found in induced sputum and BAL fluid from COPD patients [6], [7]. Adult mice over-expressing IL-1β in lung epithelium display a COPD-like phenotype consisting of lung inflammation, emphysema and airway fibrosis [8]. Elevated IL-18 levels have also been found in COPD patients and mouse models [9], [10]. Furthermore, IL-18 knockout mice show significantly decreased inflammation and emphysema compared to wild-type mice following CS exposure [11]; whereas mice over-expressing IL-18 in the lung display a COPD-like phenotype [12]. Therefore, a mechanism attenuating both cytokines may provide a substantial clinical benefit.

The NLRP3 inflammasome can be activated a number of ways; one of which is through ATP acting on the P2X7 receptor [13], [14], [15], [16]. Extracellular concentrations of ATP are maintained at low physiological concentrations by ectonucleotidases, but these concentrations increase under conditions such as infection or inflammation. This increase can be due to either greater release of ATP from cells such as epithelial or leukocytes, and/or down-regulation of ectonucleotidases [17], [18]. Recently, increases in ATP levels have been reported in *in vitro/in vivo* models of COPD [19], [20] and in clinical samples [21], [22]. This increase in ATP levels has been suggested to play a role in the chemotaxis and activation of inflammatory cells, such as neutrophils, through P2Y receptors [22], [23]. We suggest, however, that as the expression of the P2X7 receptor is increased in disease tissues/cells [22], [24] an alternative hypothesis could be that the ATP is acting on the P2X7 receptor leading to NLRP3 inflammasome and caspase 1 activation, which in turn cleaves the pro-form of IL-1β and IL-18 allowing them to be released. Indeed, Churg *et al* have recently shown that caspase 1 inhibition to attenuate a smoke driven airway inflammation [25]. These cytokines then play a central role in the inflammation observed in COPD. Our hypothesis is that modulation of this P2X7/inflammasome axis would attenuate CS-induced inflammation.

Using *in vitro* and *in vivo* pre-clinical modelling systems we show a temporal correlation between markers of the P2X7/inflammasome pathway activation and airway inflammation. We demonstrate that modulation of this pathway, using either a selective P2X7 inhibitor or P2X7 knockout mice, attenuates the airway inflammation in this *in vivo* model. This pathway, however, was not involved in the IL-1β release observed after the activation of innate host defence mechanisms triggered by an endotoxin insult. Finally, using samples from patients with COPD, we show our pre-clinical results are translational in that caspase 1 activity levels are higher in lung tissue from COPD patients and smokers compared to non-smoking donors.

## Methods

Male C57BL/6 mice were obtained from Harlan UK Limited. P2X7 receptor knockout (KO) mice (backcrossed at least 7 times) were provided by Professor Jean Kanellopoulos (Université Paris-Sud, France), originally these mice were produced in Professor Gabel's lab. For full details of how they were developed see Solle *et al*, 2001 [26]. Parallel wild type controls were bred *in-house*. The experiments were performed in accordance with the UK Home Office guidelines for animal welfare based on the Animals (Scientific Procedures) act 1986 under a project licence (PPL 70/6681).

### Development and characterisation of CS driven model of airway inflammation

 A whole body cigarette smoke exposure system consisting of a time-set pinch valve (C Lee Machining, Horsham, UK), exposure chambers (Teague Enterprises, CA, USA), extraction unit (Grainger Industrial Supply, USA) and TSP Sampling Unit (Teague Enterprises, CA, USA). Animals were exposed to either room air or cigarette smoke using 3R4F cigarettes (Cigarette filter removed, Tobacco Health Research Institute, University of Kentucky, Lexington, KY). CS is generated using a negative pressure system (flow-rate set at 1500 ml/min through the system) and timer pinch-valve to pump in smoke for pre-determined times based on the concentration of smoke required within the chambers. Room air is continuously pumped into the chamber for the remaining period between puffs. The duration of exposure periods was 50 minutes followed by a 10 minute venting period at the end where the flow is increased to maximum. Exposures for each group will take place in one of the Teague chambers (136L) chambers. A fan is placed at the bottom of the chamber on the left side where the smoke enters the chamber to ensure that the smoke is well dispersed throughout the chamber. Total suspended particulate (TSP) levels are assessed for each chamber at 15 minute intervals (15, 30, 45 minutes - 1 min sampling period) in order to validate the consistency of the smoke concentration within the chambers.

#### Dose response

 Mice were exposed to either room air (1500 ml/minute) or CS (250, 500 or 750 ml/min in a total of 1500 ml/minute) using 3R4F cigarettes (Tobacco Health Research Institute) for 50 minutes, twice daily, for three consecutive days. BAL fluid white cell counts (neutrophils, monocytes/macrophages, eosinophils and lymphocytes) were determined as previously described [27].

#### Time course

Temporal changes in airway inflammation following CS exposure (500 ml/min) were assessed (samples taken 2, 6, 24, 48, 72, 96 and 168 hours after last challenge).

Lung tissue (cytosolic fraction) caspase 1 activity levels were assessed using an assay from Enzo Life Sciences UK Limited. mRNA expression levels were measured using either Super array Custom RT^2^ Profiler PCR arrays from Tebu-Bio or real-time PCR using standard techniques and validated primers/probes [27]. Cytokines were assessed using either ultrasensitive plates from Mesoscale Discovery or standard ELISA from R&D systems.

### Development and characterisation of lipopolysaccharide (LPS) driven model of airway inflammation

Parallel experiments were performed using a stimulus of the normal innate defence system, LPS [27]. Briefly, LPS challenging was performed using a Perspex chamber (600×240×350 mm) and a System 22 nebuliser (Medic-Aid Ltd., Pagham, Sussex) driven by a high-flow-rate compressor (Medic-Aid Ltd., Pagham, Sussex). Animals were exposed to either aerosolised LPS (Escherichia coli, serotype 0111:B4, Sigma-Aldrich Ltd. Poole, UK) or endotoxin free saline (Fresenius Kabi, Warrington, UK) blown into the Perspex chamber for a 30 minute challenge period.

### Role of P2X7 receptor in CS/LPS driven airway inflammation

Wild-type and P2X7^−/−^ mice were exposed to the previously established CS or LPS exposure protocol. Twenty-four or six hours after the final exposure airway inflammation was assessed.

### Effect of P2X7 receptor antagonist on CS-induced airway inflammation

 Mice were treated with vehicle (0.5% methyl cellulose and 0.2% tween80 in distilled H_2_O, 10 ml/kg, p.o.) or A-438079, 1 hour prior to the first exposure and 1 hour after the second exposure each day. Animals were then exposed to either room air or CS as above. Airway inflammation was assessed 24 hours after the final exposure.

### Effect of P2X7 receptor antagonist on LPS induced airway inflammation

Mice were treated with vehicle or A 438079 orally, 1 hour prior to challenge. Airway inflammation was assessed 6 hours after the LPS challenge.

### Characterisation of a more chronic CS model

Male mice were exposed to room air or cigarette-smoke (500 ml/min), twice daily. Airway inflammation was determined 24 hours after 3, 7, 10, 14, 17, 21, 24 or 28 days of challenging.

### Role of P2X7 receptor in the more chronic CS driven airway inflammation

Wild-type and P2X7^−/−^ mice were exposed to room air or CS twice a day for 28 days. Twenty-four after the final exposure airway inflammation was assessed.

### Human tissue

Human lung tissue samples were obtained from a transplant programme. Informed written consent was obtained for the use of human tissues for research. Ethical approval for the study was obtained from the Royal Brompton and Harefield ethics committee (09/H0708/72).

### Compounds and materials

 AZ 11645373 was kindly provided by Astra Zeneca Pharmaceuticals. A 438079 was synthesized by Peakdale Molecular. All other agents were purchased from Sigma-Aldrich unless otherwise described.

### Data analysis

Data is expressed as mean ± s.e.m of n observations. Statistical significance was determined using either Student's t-test or One-way ANOVA followed by an appropriate post-hoc test. A P value <0.05 was taken as significant and all treatments were compared with the appropriate control group.

## Results

### Development and characterisation of CS driven model of airway inflammation

**Figure 1:**
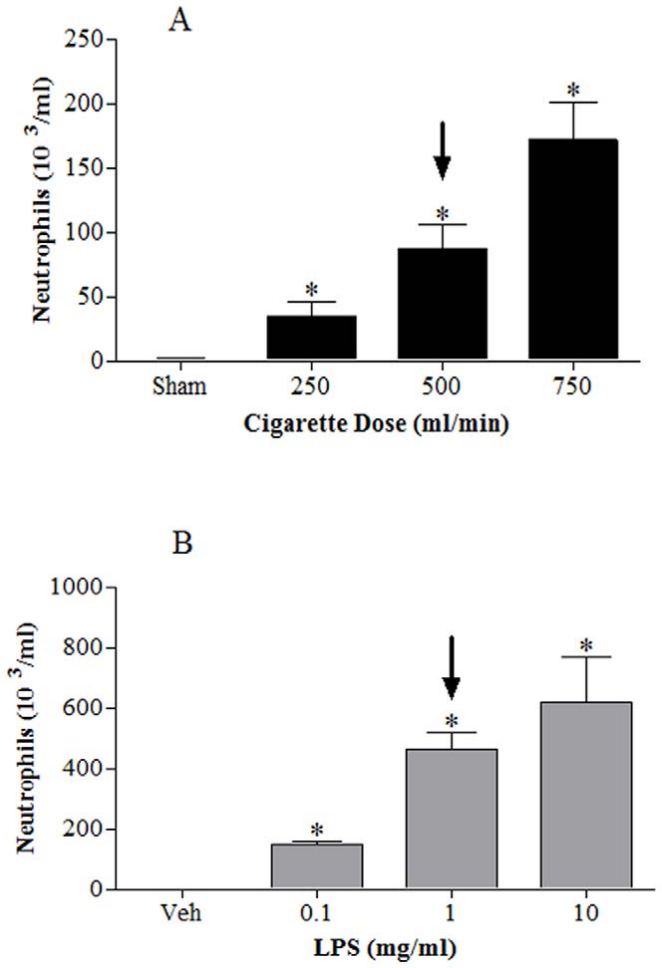
Characterisation of CS and LPS challenge models. Mice were challenged with air or CS (1 hour, twice a day, for 3 days) and lungs lavaged 24 hours after final exposure; neutrophil numbers are presented in panel **A**. Mice were challenged with aerosolised saline or LPS (30 minutes) and lungs lavaged 6 hours after exposure; neutrophil numbers are presented in panel **B**. The arrows indicate the challenge dose we chose to continue our studies. Data represent mean ± s.e.mean, n = 6. * indicates statistically significant difference from air/saline control group (One-way ANOVA with a Dunn's post test).

Exposure to CS resulted in a dose-related increase in white blood cells in the airway lumen; predominantly neutrophils (Figure 1A). The 500 ml/min dose of CS was selected as a sub-maximal stimulant dose and used throughout this project.

**Figure 2:**
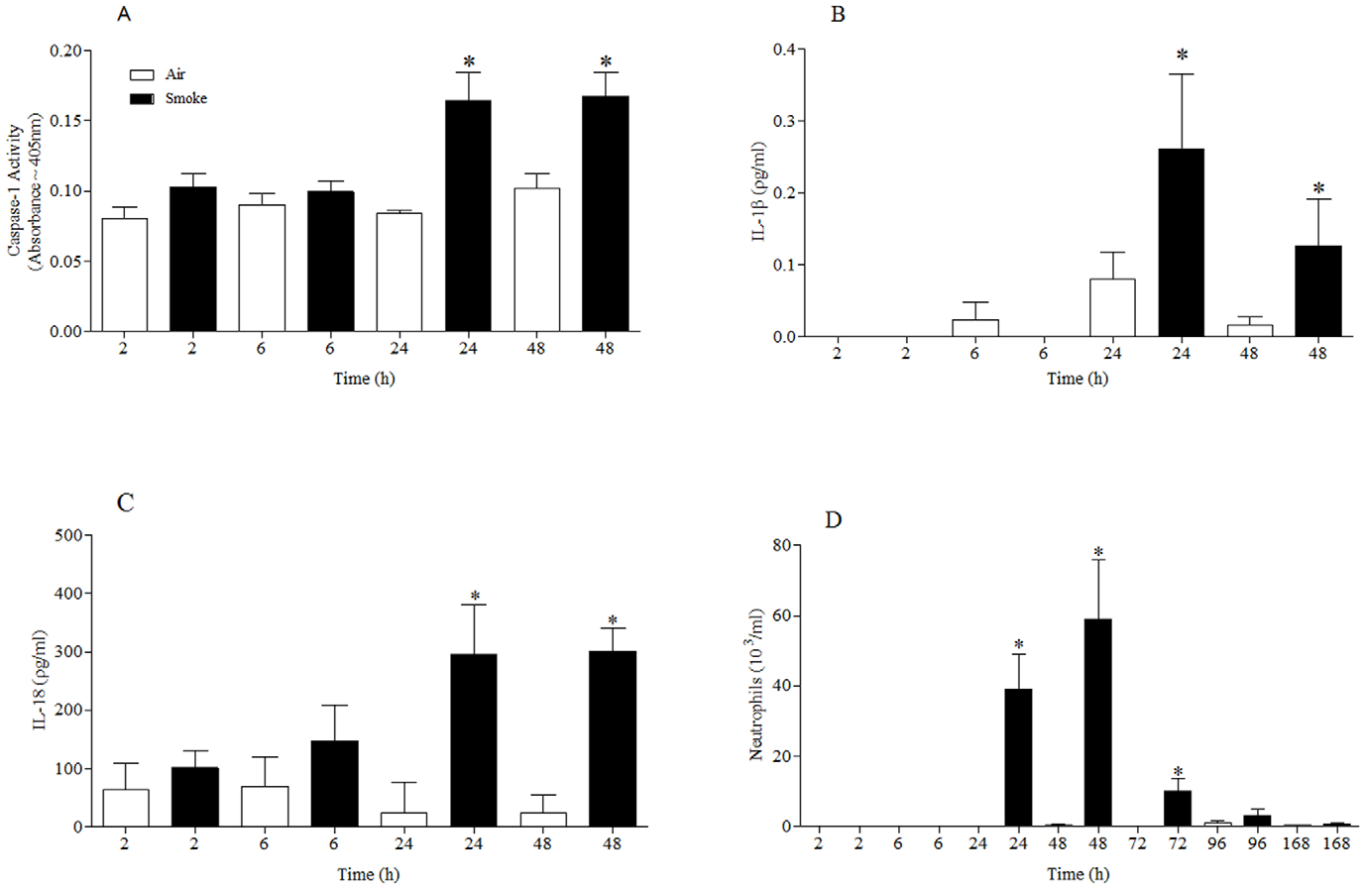
Temporal characterisation of P2X7 – NLRP3 inflammasome associated end points in the CS driven murine model. Mice were challenged with air or CS (1 hour, twice a day, for 3 days) and samples collected at various times after the last challenge. (**A**) Caspase 1 activity in the lung tissue (**B**) IL-1β levels in the BALF (**C**) IL-18 levels in the BALF (**D**) numbers of neutrophils in the BALF. Data represent mean ± s.e.mean, n = 6. * indicates statistically significant difference from time matched control group (Mann-Whitney test).

Temporal characterisation of the effects of CS exposure revealed that there appeared to be a correlation between the increase in caspase 1 activity, IL-1β/IL-18 release and airway neutrophilia (Figure 2). Caspase 1 activity in the cell free BALF was not altered (data not shown). Further analysis showed that CS challenge did not greatly alter lung tissue levels of IL-1β, IL-18, pro-caspase 1 and P2X7 mRNA (Text S1; Figure S1).

### Development and characterisation of LPS driven model of airway inflammation

 Exposure to endotoxin, a trigger of the innate host defence mechanism, resulted in a dose-related increase in white blood cells in the airway lumen; again the profile of cell type increased was predominantly neutrophilia (Figure 1). The 1 mg/ml dose of LPS was selected as a sub-maximal stimulant dose and used throughout this project.

**Figure 3:**
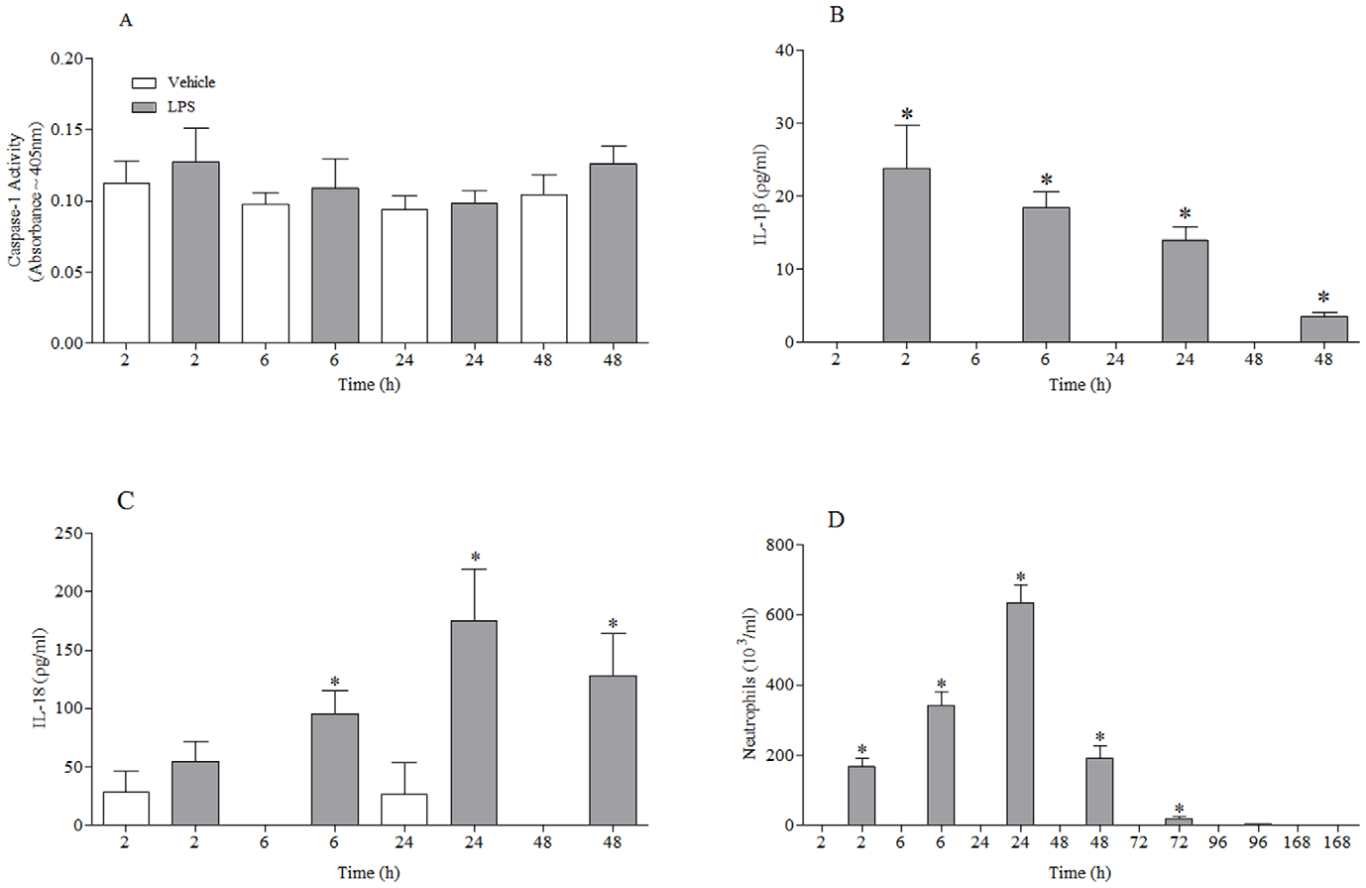
Temporal characterisation of P2X7 – NLRP3 inflammasome associated end points in the LPS driven murine model. Mice were challenged with vehicle (endotoxin free sterile saline for 30 minutes) or LPS (1 mg/ml) and samples collected at various times after the last challenge (**A**) Caspase 1 activity in the lung tissue (**B**) IL-1β levels in the BALF (**C**) IL-18 levels in the BALF (**D**) numbers of neutrophils in the BALF. Data represent mean ± s.e.mean, n = 6. * indicates statistically significant difference from time matched control group (Mann-Whitney test).

 Temporal characterisation of the effects of LPS exposure revealed that there appeared to be a correlation between the increase in IL-1β/IL-18 release and airway neutrophilia (Figure 3), however unlike the CS model there was no increase in caspase 1 activity. Caspase 1 activity in the cell free BALF was not altered (data not shown). Further analysis showed that mRNA levels of pro-caspase 1 and IL-1β increased dramatically in the lung tissue after LPS challenge (Text S1; Figure S2). Whereas IL-18 and P2X7 mRNA levels were either not altered or decreased (Figure S2).

### Role of P2X7 receptor in CS driven airway inflammation

**Figure 4:**
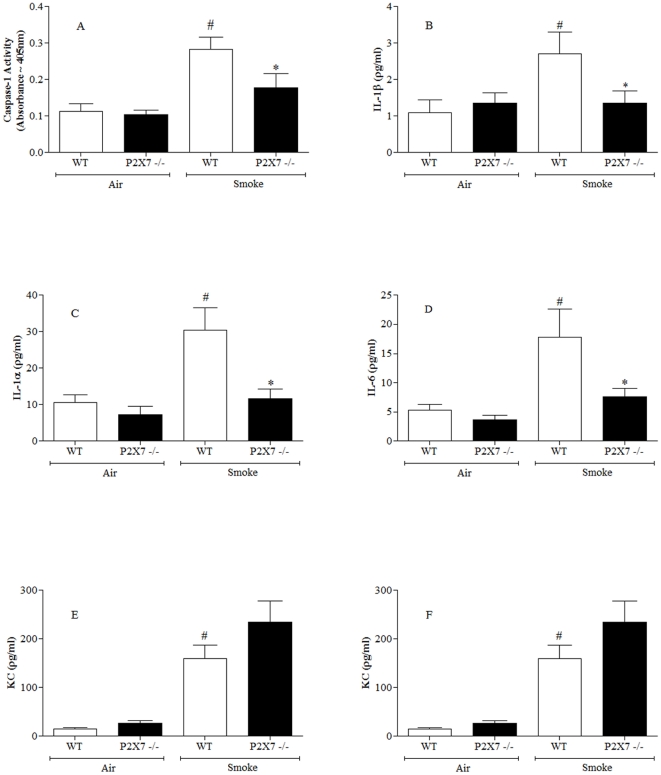
Role of the P2X7 – NLRP3 inflammasome axis in the CS driven murine model. Wild type or P2X7 knockout mice were challenged with air or CS and samples collected 24 hours after the last challenge. (**A**) Caspase 1 activity in the lung tissue (**B**) IL-1β levels in the BALF (**C**) IL-1α levels in the BALF (**D**) IL-6 levels in the BALF (**E**) KC levels in the BALF (**F**) numbers of neutrophils in the BALF. Data represent mean ± s.e.mean, n = 8. # indicates statistically significant difference from air challenge control group. * indicates statistically significant difference from CS challenged control group (Mann-Whitney test).

Having established there was temporal correlative data to suggest a role for the P2X7-inflammasome pathway in CS driven inflammation, we further investigated its role by comparing wild-type and P2X7^−/−^ mice. As can be seen in Figure 4, CS exposure failed to increase caspase 1 activity, IL-1β and airway neutrophilia in P2X7^−/−^ mice. This is strong evidence for a role of the P2X7-inflammasome axis in CS-induced airway neutrophilia. Interestingly, levels of IL-1α and IL-6 were also attenuated in the knockout mice, whereas KC levels were not altered (Figure 4).

### Role of P2X7 receptor in LPS driven airway inflammation

**Figure 5:**
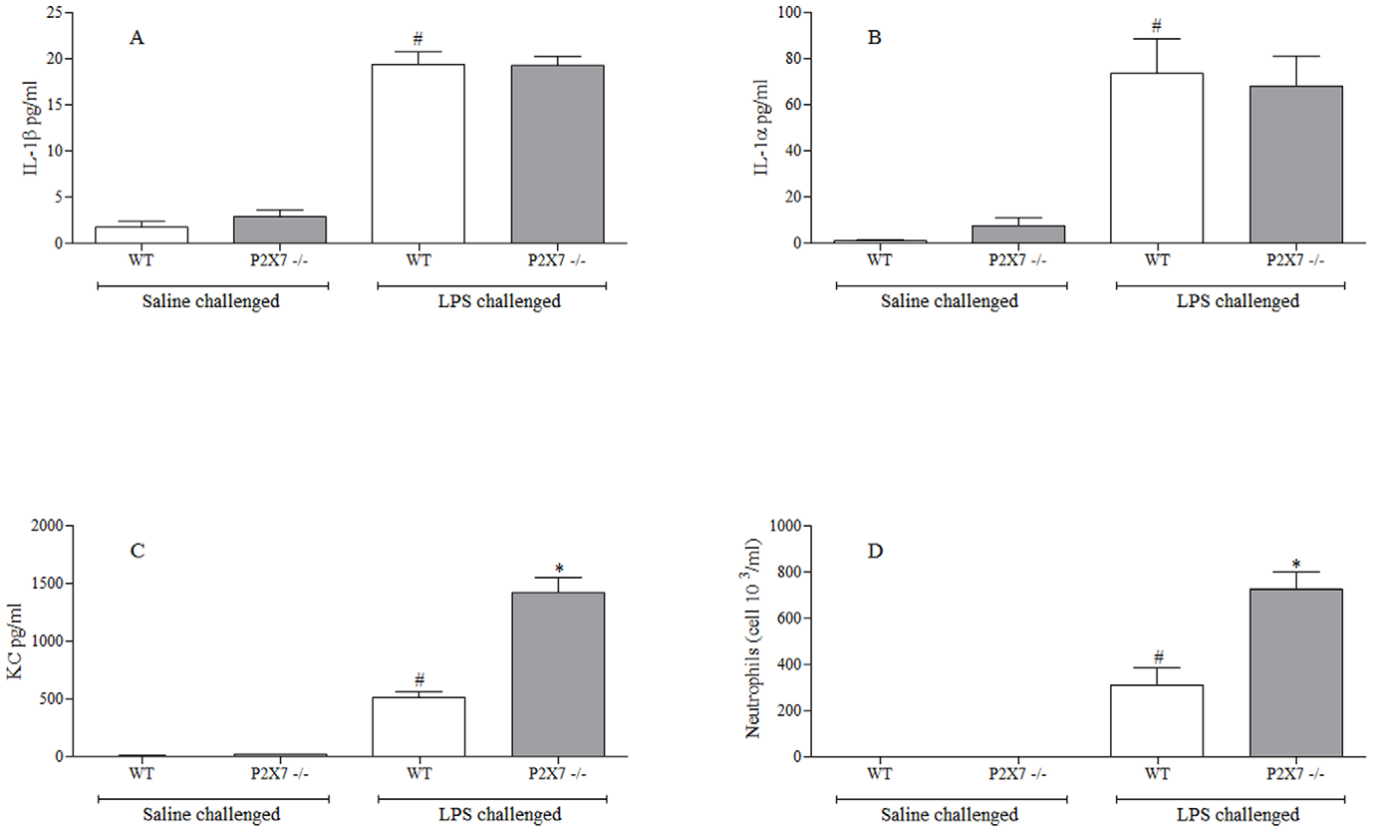
Role of the P2X7 – NLRP3 inflammasome axis in the LPS driven murine model. Wild type or P2X7 knockout mice were challenged with saline or LPS and samples collected 6 hours after the last challenge. (**A**) IL-1β levels in the BALF (**B**) IL-1α levels in the BALF (**C**) KC levels in the BALF (**D**) numbers of neutrophils in the BALF. Data represent mean ± s.e.mean, n = 8. # indicates statistically significant difference from air challenge control group. * indicates statistically significant difference from CS challenged control group (Mann-Whitney test).

Figure 3 indicated that in the endotoxin driven inflammation there was not a role for the P2X7-inflammasome axis. To investigate this further wild-type and P2X7^−/−^ mice were challenged with LPS. As expected P2X7^−/−^ mice did not have altered levels of the inflammasome linked IL-1β, or indeed IL-1α (Figure 5). Interestingly, we did observe a statistical significant increase in BAL KC levels and neutrophilia (Figure 5).

### Establishing optimum P2X7 inhibitor

To confirm the data with the P2X7^−/−^ mice we wanted to parallel the study using a selective small molecular weight receptor inhibitor. Prior to doing this we needed to establish an appropriate tool to use in our murine model. To do this we first selected appropriate human and mouse cells to develop a P2X7-inflammasome driven *in vitro* assay. We detected P2X7 receptor and pro-caspase 1 expression at the mRNA and protein level in human (THP-1) and mouse (J774) monocytes/macrophages (Text S1; Figure S3). Using these cell types we established sub-maximal concentrations of ATPγS (P2X7 agonist) or LPS that induced IL-1β release (data not shown). Combination of these two stimuli resulted in enhanced release of inflammasome linked cytokines, IL-1β and IL-18, but not other cytokines such as TNFα and IL-6 (data not shown). Using these systems we determine the impact of 2 structurally distinct P2X7 inhibitors. The combination of ATPγS and LPS leads to enhanced IL-1β release (Text S1; Figure S4). Treatment with AZ 11645373 attenuated the enhanced release of IL-1β in human cells, suggesting successful blockade of the human P2X7 receptors, but not murine cells (Text S1; Figure S4). Conversely, A 438079 failed to have an impact in the human cell assay but did block the enhanced release of IL-1β from murine cells (Text S1; Figure S4). A 438079 did not alter levels of TNFα or IL-6 (data not shown). We did not detect any IL-1α release in either of the cell systems (data not shown).

### Effect of P2X7 receptor antagonist on CS/LPS-induced airway inflammation

**Figure 6:**
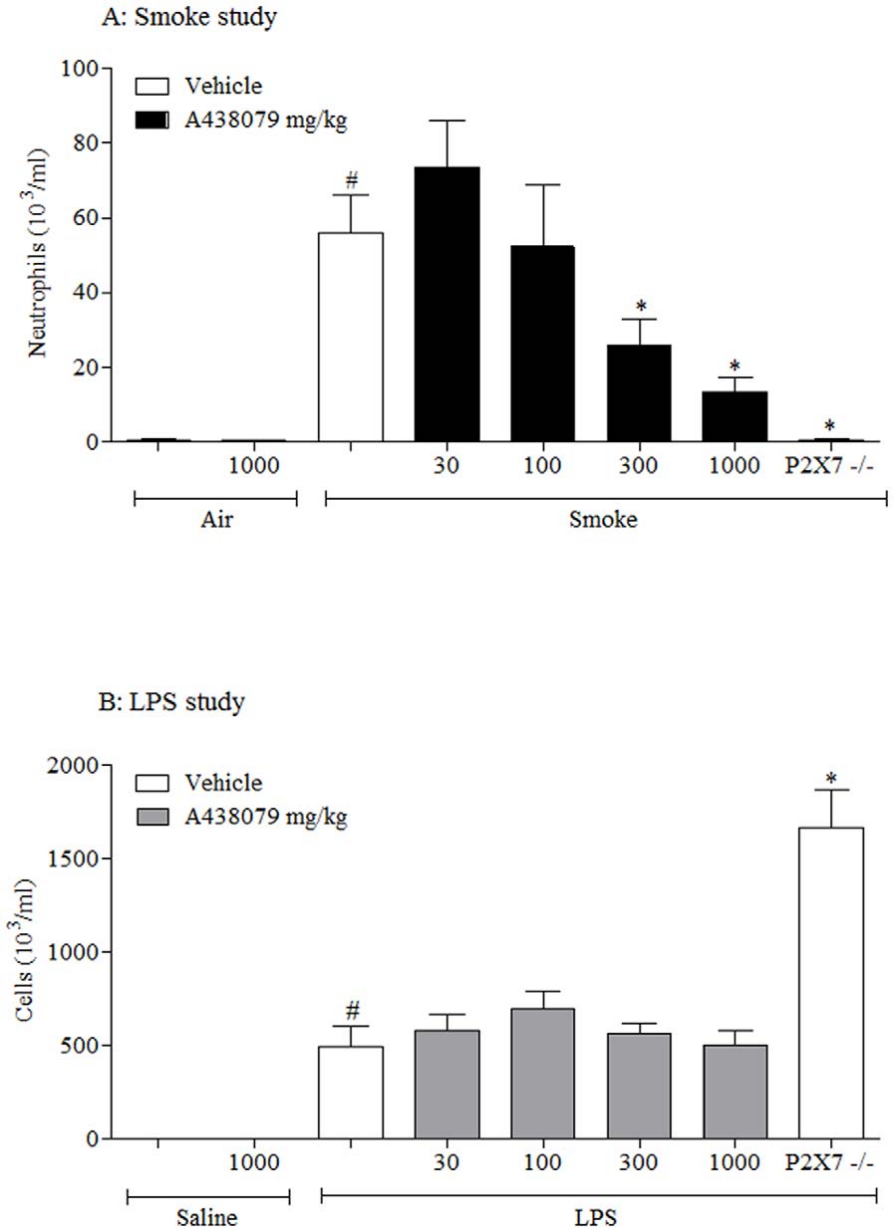
Role of the P2X7 – NLRP3 inflammasome axis in the CS or LPS driven murine models. (**A**) Wild type or P2X7 −/− mice were challenged with air or CS and received vehicle (0.5% methyl cellulose and 0.2% tween80 in distilled water, 10 ml/kg, orally) or A 438079 twice a day and one hour prior to the cull. Numbers of neutrophils in the airway lumen was measured twenty-four hours after the final challenge. (**B**) Wild type or P2X7 −/− mice were challenged with saline or LPS and received vehicle or A 438079 1 hour prior to challenge. Numbers of neutrophils in the airway lumen was measured six hours after the challenge. Data represent mean ± s.e.mean, n = 8. # indicates statistically significant difference from air/saline challenge control group. * indicates statistically significant difference from CS/LPS challenged control group (One-way ANOVA with a Dunn's post test).

 Having selected an appropriate tool to block murine P2X7 receptors we profiled it in the CS driven model. Consistent with the P2X7^−/−^ mice, the compound caused a dose related inhibition in airway neutrophilia (Figure 6A). Lymphomononuclear cell numbers: Air/Vehicle−78.0±5.9; CS/Vehicle−119.4±11.7; CS/compound (1000 mg/kg)−138.9±9.3; CS/P2X7 −/− −120.9±10.9 cell 10^3^/ml.

 In the LPS driven model, unlike in the P2X7^−/−^ mice, the compound had no impact on neutrophil levels (Figure 6B). Lymphomononuclear cell numbers: Saline/Vehicle−69.3±5.2; LPS/Vehicle−38.9±6.4; LPS/compound (1000 mg/kg)−34.1±3.6; LPS/P2X7 −/− −34.1±7.3 cell 10^3^/ml.

### Characterisation of a more chronic CS model

**Figure 7:**
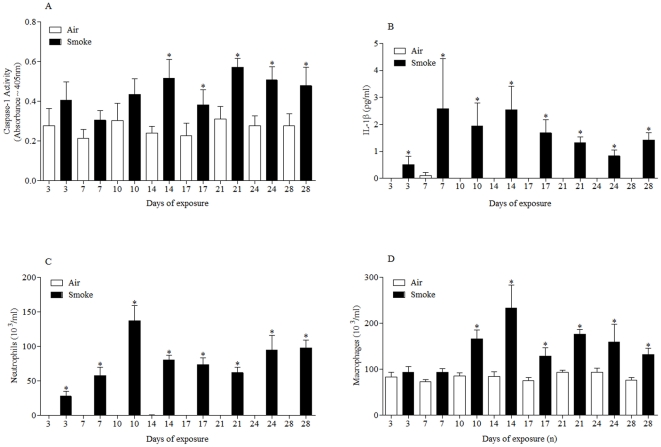
Temporal characterisation of P2X7 – NLRP3 inflammasome associated end points in the sub-chronic CS driven murine model. Mice were challenged with air or CS (1 hour, twice a day, for 3–28 days) and lungs lavaged 24 hours after final exposure. **A**) Caspase 1 activity in the lung tissue (**B**) IL-1β levels in the BALF (**C**) numbers of neutrophils in the BALF (**D**) numbers of macrophages in the BALF. Data represent mean ± s.e.mean, n = 6. * indicates statistically significant difference from time matched control group (Mann-Whitney test).

The data so far strongly suggested that the P2X7-inflammasome axis is central to the inflammation induced after CS exposure. To determine if this axis is important in ongoing inflammation, we measured markers of the pathway in a more chronic model. Temporally correlated with the increase in neutrophils and macrophages observed (Figure 7), we measured an increase in caspase 1 activity and IL-1β levels. This suggests that the P2X7-inflammasome axis is not only central to the induction of CS-induced inflammation but also in the ongoing inflammation.

### Role of P2X7 receptor in a more chronic CS driven airway inflammation

**Figure 8:**
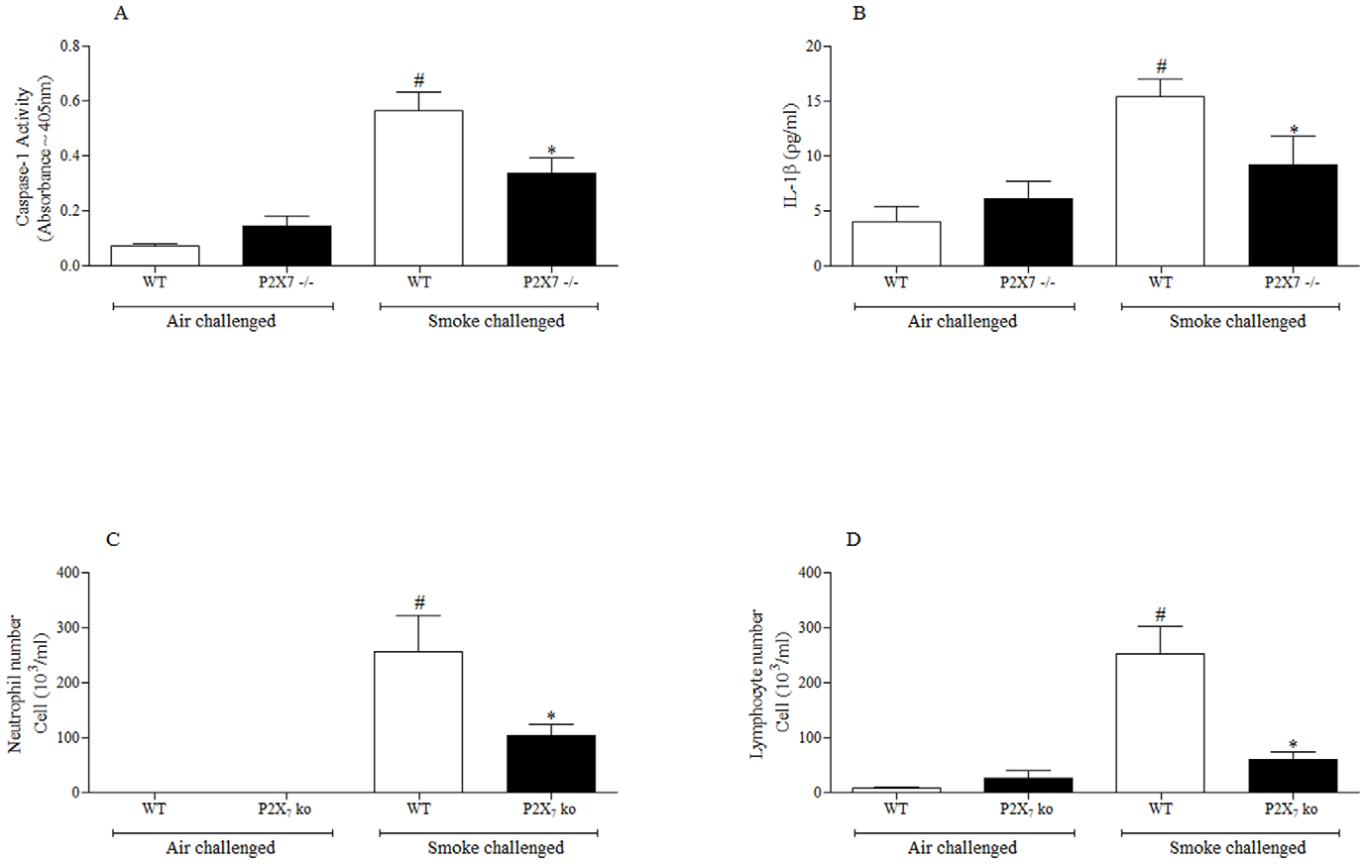
Role of the P2X7 – NLRP3 inflammasome axis in the sub-chronic CS driven murine model. Wild type or P2X7 knockout mice were challenged with air or CS twice a day for 28 days and samples collected 24 hours after the last challenge. (**A**) Caspase 1 activity in the lung tissue (**B**) IL-1β levels in the BALF (**C**) numbers of neutrophils in the BALF (**D**) numbers of lymphocytes in the BALF. Data represent mean ± s.e.mean, n = 5–8. # indicates statistically significant difference from air challenge control group. * indicates statistically significant difference from CS challenged control group (Mann-Whitney test).

To further investigated the P2X7-inflammasome axis in the more chronic model we compared the inflammation in wild-type and P2X7^−/−^ mice after 28 days of CS exposure. As can be seen in Figure 8 P2X7^−/−^ mice had significantly reduced caspase 1 activity, BAL IL-1β, neutrophils and lymphocytes. Macrophages numbers were also reduced (data not shown).

### Expression of P2X7-inflammasome axis in human tissue

**Figure 9:**
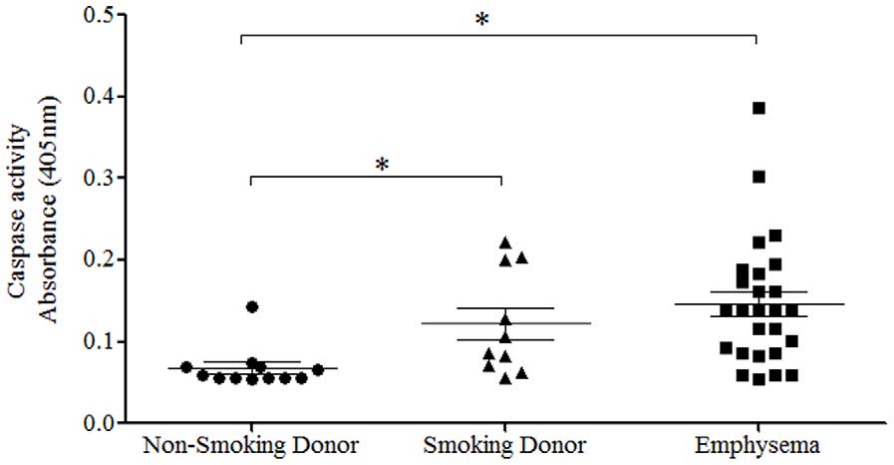
Caspase 1 activity levels in human lung samples. Lung samples were collected from transplant surgery either donor tissue (non-smokers or smokers) or diseased tissue. Caspase 1 activity in the cytoplasmic fraction of the extracted lung tissue was assessed using a specific assay. Data represent as individual data with bar indicating mean ± s.e.mean. * indicates statistically significant difference from non-smoking donor lung tissue levels (One-way ANOVA with a Dunn's post test).

To determine if our observations in the pre-clinical models translated into the human disease, we measured caspase 1 activity in lung tissue from non-smoking donors, smoking donors and emphysema patients (details of which are listed in Table 1). Although the sample numbers and patient information are limited, it can be clearly seen that, like in our pre-clinical models, caspase 1 activity is increased in diseased tissue (Figure 9).

**Table 1:** Details on the donor/recipient.

Disease Group	Age (years)	Sex (M/F)
Non-Smoking Donor	27–72	3/9
Smoking Donor	22–62	7/3
Emphysema	49–69	17/8

## Discussion

COPD is a progressive condition associated with exposure to CS and is currently reported to be the fourth leading cause of death worldwide and is predicted to rise to the third [3]. Furthermore, COPD-related costs outstrip those of other respiratory diseases such as asthma and as yet there are currently no pharmacological therapies to combat the pathogenesis and relentless progression of this disease [5]. Understanding the mechanisms driving the chronic inflammation seen in patients with the disease would undoubtedly aid the development of effective medication. In the inflammatory *milieu* present in the lungs of smoke-exposed mice, and human patients with COPD, are increased levels of cytokines linked to the activation of the NLRP3 inflammasome i.e. IL-1β and IL-18 [6], [7], [8], [9], [10], [28]. There is some evidence to suggest that these cytokines are central to the inflammation seen in models of COPD [11], [29]. Recently it has been shown that levels of an activator of the NLRP3 inflammasome, ATP, are increased in pre-clinical smoke-exposure models [23], [24]. Additionally, increased ATP levels in the lungs of patients with COPD have been shown to be associated with a decline in lung function and an increase in inflammatory cellular burden [21]. We suggest that exposure to CS leads to the release of endogenous danger signals (e.g. ATP) which activates the NLRP3 inflammasome via the P2X7 purinergic receptor [13] and thus causes the maturation and subsequent release of IL-1β and IL-18. We hypothesise that blockade of the P2X7 – NLRP3 inflammasome pathway will attenuate the inflammation present in CS-induced airway inflammation. Through the use of *in vitro* and *in vivo* pre-clinical modelling systems we showed a temporal correlation between markers of the P2X7/inflammasome pathway activation and airway inflammation. Furthermore, we demonstrate that modulation of this pathway, using either a selective P2X7 inhibitor or P2X7 knockout mice, attenuates the airway inflammation in this disease model. This data is consistent with data recently published by Lucattelli *et al* [24]. This pathway, however, was not involved in a similar inflammatory response (i.e. IL-1β release) observed after the normal innate defence mechanism is triggered by an endotoxin insult suggesting that this pathway is only activated in disease settings. Finally, using lung samples collected from patients with COPD, we demonstrate that markers of inflammasome activation are evident in tissue from diseased patients with COPD.

Exposure to CS, whether it be acute (induction of the inflammation) or more chronic (up to 28 days), resulted in an increase in caspase 1 activity and release of NALP-3 inflammasome linked cytokine IL-1β. A similar increase in caspase 1 activity was observed in lung tissue from patients with COPD so severe/chronic that they required a lung transplant which suggests that the pre-clinical model reflects the clinical disease and that the role of caspase 1 is not restricted to the induction of the inflammation but is chronically elevated. We postulate that the increase in activity was not due to an increased amount of caspase 1 because we did not observe an increase in mRNA expression. On caveat is that the increase in caspase activity observed in the human lung samples may not be through activation of the P2X7 receptor as other things (like uric acid or reactive oxygen species) than can activate NLRP3. Further assessment of the CS models revealed no change in IL-1β mRNA levels which is intriguing because we did measure an increase in this cytokine at the protein level. As the mechanism of action of glucocorticoids is generally thought to be blockade of transcription/translation of inflammatory cytokines, this could be one reason why the inflammation observed in COPD patients is resistant to glucocortoid treatment. If the inflammation is driven via a mechanism which does not rely on transcription/translation it could explain the limited impact of a steroid treatment.

Further evidence for a central role of the P2X7 – NLRP3 inflammasome pathway in CS-induced neutrophilia came from the studies we performed with either mice missing functional P2X7 receptors or a small molecular weight inhibitor. The fact that we obtained very similar data using both mechanisms of blocking the P2X7 receptor makes the results more compelling. It was interesting to note that cytokines not thought to be linked to the inflammasome/caspase 1 were also reduced after blockade of the P2X7 receptor. We suggest that the reduction in IL-1α and IL-6 could be a function of blocking the neutrophilia, rather than a direct effect on the production/release of the cytokines themselves. Indeed in the cell based systems cytokines such as IL-6 and TNFα were not affected by the P2X7 inhibitor. We could not detect any IL-1α release in our human and mouse cell based systems which, although possibly different to that reported by others [30], would indicate further that this cytokine is not controlled by the mechanisms which govern IL-1β/IL-18 release. IL-1α release has been suggested to be controlled by IL-1β, thus it could be that the inhibition of IL-1β release accounts for the reduction we observed in IL-1α [31]. The observation that the levels of KC were not affected by blockade of the P2X7–inflammasome axis is worthy of note. It would suggest that the CS-induced production/release of KC is via another mechanism, its source being cells present in the lung, rather than the neutrophils being recruited.

To establish an appropriate pharmacological P2X7 tool to use in the murine models we used human and murine cell based assays. We selected the cell type based on the expression of targets of interest i.e. the P2X7 receptor and caspase 1 and from the literature [26], [32]. We found that human THP-1 cells and murine equivalent J774 cells expressed both targets at the mRNA and protein level, whereas the epithelial cell lines (A549 and LA4) did not which is consistent to that reported by other groups [31]. This would suggest that the inflammasome-linked cytokines in the airways are more likely to be produced by macrophages/monocytes rather than epithelial cells. When we stimulated the THP-1/J774 cells with both LPS and ATP the level of IL-1β release was greater than the sum of the stimuli separately. Using these modelling systems we demonstrated that the P2X7 receptor was central to this enhanced release of IL-1β; this has been demonstrated by many other groups [16], [26], [32], [33], [34]. Of interest to us was that AZ 11645373 failed to impact on the murine cells, whereas A 438079 only impacted on the murine system, even though it is reported to act on the human receptor [35]. Despite the apparent lack of activity in the human cell system, we were able to use A 438079 as a tool in our murine models.

Interestingly although IL-1β (and IL-1α) levels after LPS challenge was not altered in the P2X7 −/− mice, we did observe an increase in airway KC and neutrophilia. The fact that we did not parallel this increase with the P2X7 inhibitor may indicate that the phenomenon is related to either the genetic manipulation or long term/complete blockade of this receptor.

In conclusion these results advocate the critical role of the P2X7/inflammasome axis in cigarette smoke induced inflammation. What is more the data suggests that the pathway has an ongoing role in the pathogenesis of COPD and highlights it as possible therapeutic targets in combating this deadly disease.

## Supporting Information

Figure S1 **Temporal characterisation of P2X7 – NLRP3 inflammasome associated end points in the CS driven murine model.** Mice were challenged with air or CS (1 hour, twice a day, for 3 days) and samples collected at various times after the last challenge. (**A**) IL-1β mRNA levels in the lung tissue (**B**) IL-18 mRNA levels in the lung tissue (**C**) caspase 1 mRNA levels in the lung tissue (**D**) P2X7 mRNA levels in the lung tissue. Data represent mean ± s.e.mean, n = 6. * indicates statistically significant difference from time matched control group (Mann-Whitney test).
(TIF)

Figure S2 **Temporal characterisation of P2X7 – NLRP3 inflammasome associated end points in the LPS driven murine model.** Mice were challenged with saline or LPS (30 minutes) and samples collected at various times after the last challenge. (**A**) IL-1β mRNA levels in the lung tissue (**B**) IL-18 mRNA levels in the lung tissue (**C**) caspase 1 mRNA levels in the lung tissue (**D**) P2X7 mRNA levels in the lung tissue. Data represent mean ± s.e.mean, n = 6. * indicates statistically significant difference from time matched control group (Mann-Whitney test).
(TIF)

Figure S3 **Caspase 1 and P2X7 mRNA levels in a range of human and mouse cell type.** Cells were collected after standard culture conditions, mRNA extracted and RT-PCR performed to establish mRNA expression levels of caspase 1 and P2X7. Caspase 1 mRNA levels in human and murine cells are shown in A and C, respectively. P2X7 mRNA levels in human and murine cells are shown in B and D, respectively. Data represent mean ± s.e.mean, n = 3–6.
(TIF)

Figure S4 **Determining the effectiveness of P2X7 receptor antagonist using human and mouse monocytes.** Cultured cells were exposed to either sub-maximal concentration of ATPγs (1 mM) or LPS (0.1 µg/ml) or a combination of the two. Cells were pre-treated with vehicle or one of the inhibitors; IL-1β levels were measured by ELISA. (**A**) Effect of AZ 11645373 in THP-1 cells (**B**) Effect of AZ 11645373 in J774 cells (**C**) Effect of A 438079 in THP-1 cells (**D**) Effect of A 438079 in J774 cells. Data represent mean ± s.e.mean, 3 experimental runs each with n = 2. * indicates statistically significant difference from the respective control group (Mann-Whitney test and one-way ANOVA followed by a Dunn's post test).
(TIF)

Text S1 (DOCX)
