# Supplementary figures and images for: P2X7 Receptor and Caspase 1 Activation Are Central to Airway Inflammation Observed after Exposure to Tobacco Smoke
Source: PLoS One. 2011 Sep 6;6(9):e24097. doi: 10.1371/journal.pone.0024097 (PMC3167831; doi:10.1371/journal.pone.0024097)

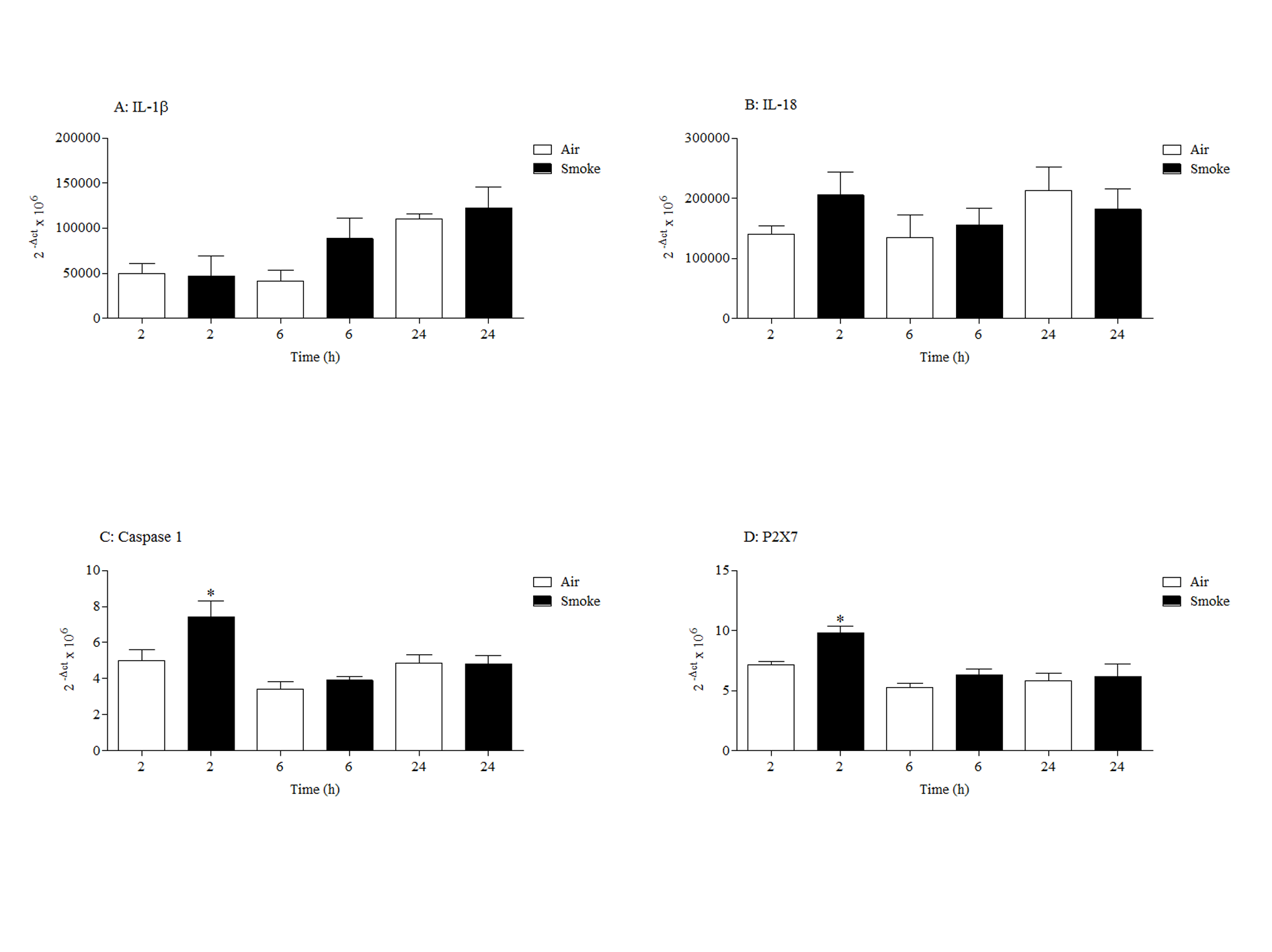

Supplement: Figure S1 — Temporal characterisation of P2X7 – NLRP3 inflammasome associated end points in the CS driven murine model. Mice were challenged with air or CS (1 hour, twice a day, for 3 days) and samples collected at various times after the last challenge. (A) IL-1β mRNA levels in the lung tissue (B) IL-18 mRNA levels in the lung tissue (C) caspase 1 mRNA levels in the lung tissue (D) P2X7 mRNA levels in the lung tissue. Data represent mean ± s.e.mean, n = 6. * indicates statistically significant difference from time matched control group (Mann-Whitney test). (TIF) [file pone.0024097.s001.tif]

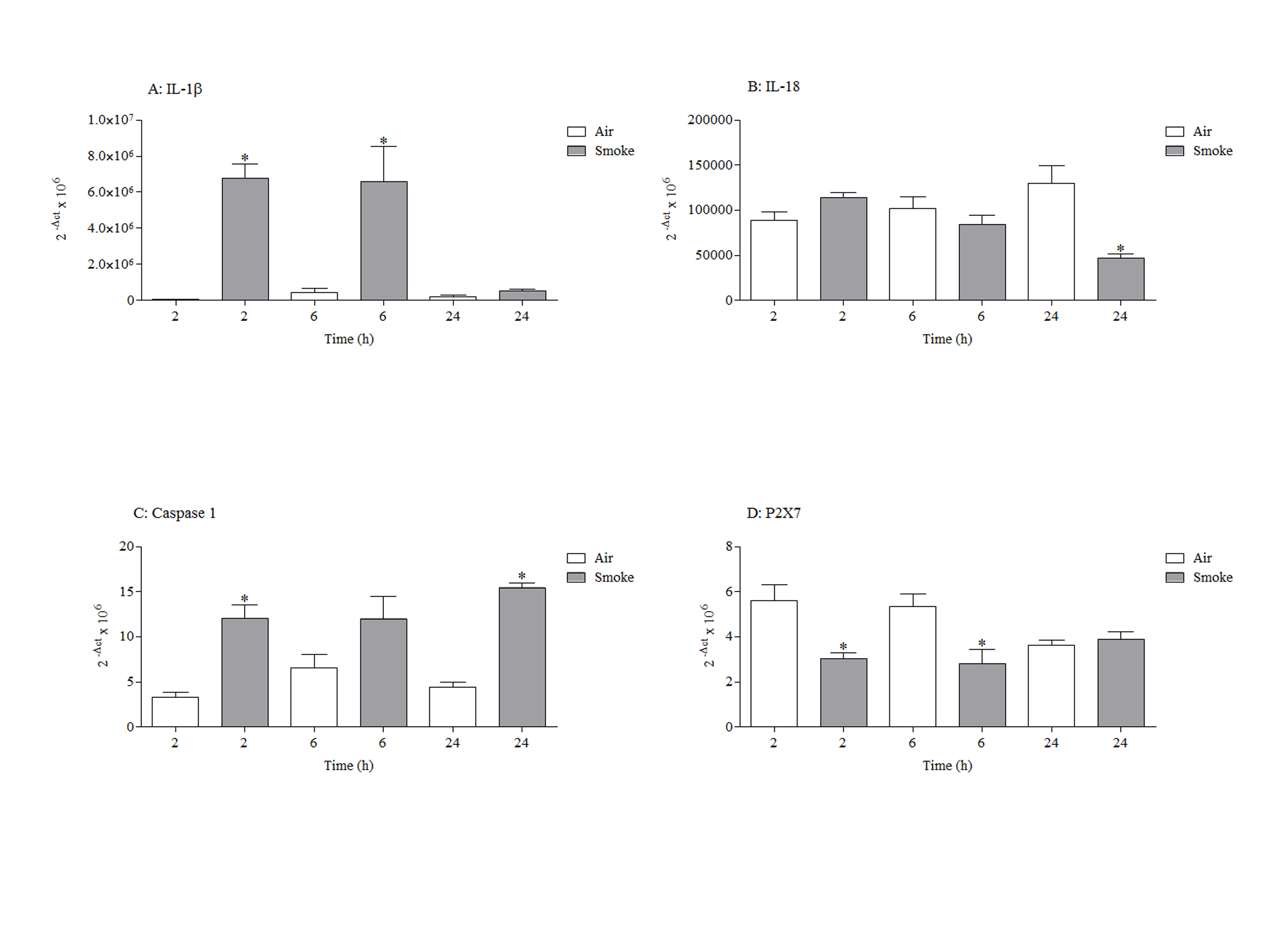

Supplement: Figure S2 — Temporal characterisation of P2X7 – NLRP3 inflammasome associated end points in the LPS driven murine model. Mice were challenged with saline or LPS (30 minutes) and samples collected at various times after the last challenge. (A) IL-1β mRNA levels in the lung tissue (B) IL-18 mRNA levels in the lung tissue (C) caspase 1 mRNA levels in the lung tissue (D) P2X7 mRNA levels in the lung tissue. Data represent mean ± s.e.mean, n = 6. * indicates statistically significant difference from time matched control group (Mann-Whitney test). (TIF) [file pone.0024097.s002.tif]

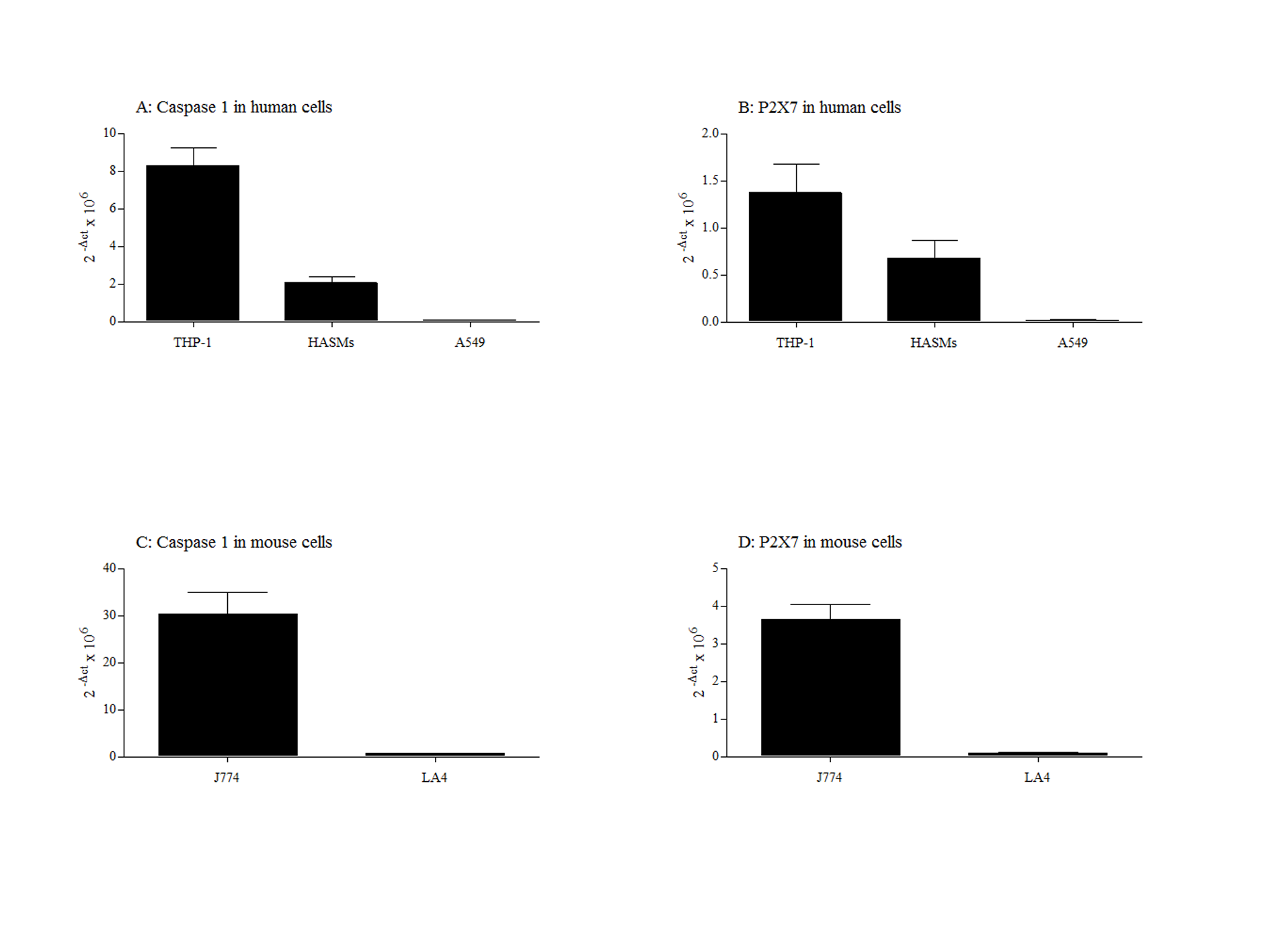

Supplement: Figure S3 — Caspase 1 and P2X7 mRNA levels in a range of human and mouse cell type. Cells were collected after standard culture conditions, mRNA extracted and RT-PCR performed to establish mRNA expression levels of caspase 1 and P2X7. Caspase 1 mRNA levels in human and murine cells are shown in A and C, respectively. P2X7 mRNA levels in human and murine cells are shown in B and D, respectively. Data represent mean ± s.e.mean, n = 3–6. (TIF) [file pone.0024097.s003.tif]

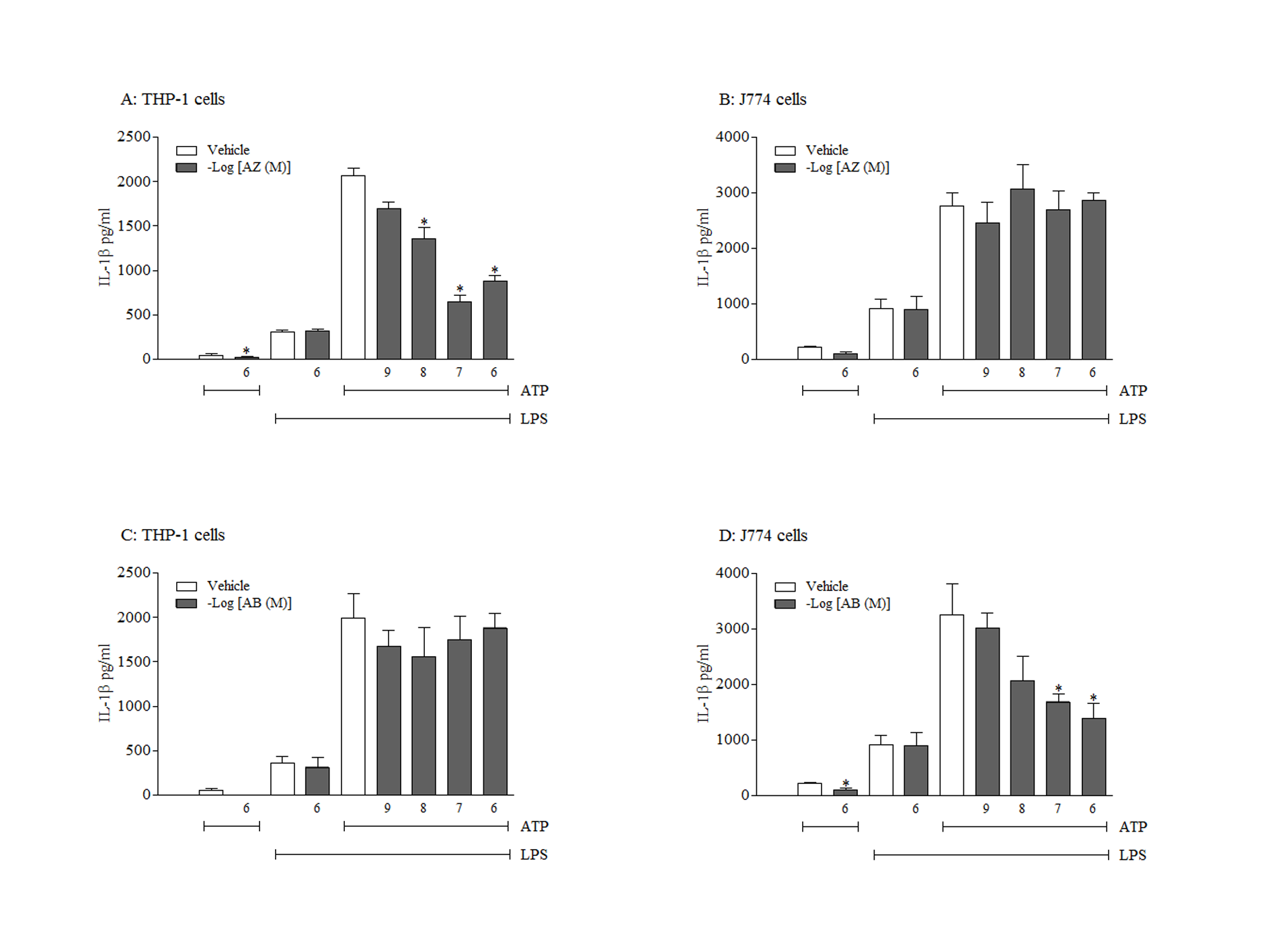

Supplement: Figure S4 — Determining the effectiveness of P2X7 receptor antagonist using human and mouse monocytes. Cultured cells were exposed to either sub-maximal concentration of ATPγs (1 mM) or LPS (0.1 µg/ml) or a combination of the two. Cells were pre-treated with vehicle or one of the inhibitors; IL-1β levels were measured by ELISA. (A) Effect of AZ 11645373 in THP-1 cells (B) Effect of AZ 11645373 in J774 cells (C) Effect of A 438079 in THP-1 cells (D) Effect of A 438079 in J774 cells. Data represent mean ± s.e.mean, 3 experimental runs each with n = 2. * indicates statistically significant difference from the respective control group (Mann-Whitney test and one-way ANOVA followed by a Dunn's post test). (TIF) [file pone.0024097.s004.tif]
