# Supplementary material for: P2X7 Receptor and Caspase 1 Activation Are Central to Airway Inflammation Observed after Exposure to Tobacco Smoke
Source: PLoS One. 2011 Sep 6;6(9):e24097. doi: 10.1371/journal.pone.0024097 (PMC3167831; doi:10.1371/journal.pone.0024097)
Supplement: Text S1 — (DOCX) [file pone.0024097.s005.docx]

**Supplementary information S1**

**Methods:** Male C57BL/6 mice were obtained from Harlan UK Limited. P2X7 receptor knockout (KO) mice (back crossed at least 7 times) were provided by Professor Jean Kanellopoulos (Université Paris-Sud, France) and parallel wild type controls were bred *in-house*. The experiments were performed in accordance with the UK Home Office guidelines for animal welfare based on the Animals (Scientific Procedures) act 1986 under a project licence (PPL 70/6681).

**Characterisation of CS driven model of airway inflammation.**

Mice were exposed to either room air (1500 ml/minute) or cigarette smoke (500 ml/min in a total of 1500 ml/minute) using 3R4F cigarettes (Tobacco Health Research Institute) for 50 minutes, twice daily, for three consecutive days. Temporal changes in airway inflammation were assessed (lung tissue samples taken 2, 6 or 24 hours after last challenge). mRNA expression levels in the lung tissue were measured using either Super array Custom RT^2^ Profiler PCR arrays from Tebu-Bio or real-time PCR using standard techniques.

**Characterisation of LPS driven model of airway inflammation.**

Mice were exposed to either saline (30 minutes) or LPS (1 mg/ml), temporal changes in airway inflammation were assessed (lung tissue samples taken 2, 6 or 24 hours after last challenge). mRNA expression levels were measured in the lung tissue using either Super array Custom RT^2^ Profiler PCR arrays from Tebu-Bio or real-time PCR using standard techniques.

**Characterising a P2X7 inhibitor in cell based assays to determine its use as a tool compound.**

Caspase 1 and P2X7 mRNA expression levels, under normal tissue culture conditions, were determined in a range of human and mouse cell types using real-time PCR using standard techniques and validated assays. Human (THP-1) and mouse (J774) monocytes/macrophages were selected to establish the efficacy of the P2X7 inhibitors – AZ 11649373 and A 438079. Both cell lines were purchased from the European Collection of Cell Cultures (ECACC, Salisbury, Wiltshire, UK) and the frozen ampoule was left at room temperature for approximately 1 minute and then transferred to a 37°C water bath for 1-2 minutes until fully thawed. The cells were then cultured in RPMI 1640 with glutamax I (Invitrogen Ltd, UK) supplemented with 10% FCS and 1% antibiotic and antimycotic solution (Penicillin/Streptomycin – Sigma-Aldrich Co., Poole, UK) at 37°C in a humidified atmosphere (95% air, 5% (v/v) CO_2_). They were cultured into 75cm^3^ flasks, and the media was replaced after 3 days and thereafter every 48 hours. The J774.2 cell line must be scraped from the bottom of the flask before replacing the media. The media was changed by centrifuging the cell suspension at 800 x g for 5 minutes at room temperature, in a centrifuge (Mistrall 3000i, MSE). The supernatant was discarded, and the pellet of cells was resuspended in 1ml of RPMI 1640 with glutamax I, supplemented with 10% FCS and 1% antibiotic and antimycotic solution. Trypan Blue exclusion was performed to determine cell viability and cells were passaged into 2 x 75 cm^3^ flasks when cell numbers reached 10 x 10^6^ cells/ml. Both cell lines have a doubling time of approximately 48 hours.

One experimental day’s 400,000 cells were added to each well in a 24 well plate. The cells were then treated (vehicle or sub-maximal concentrations of LPS (0.1 μg/ml) and/or ATPγS (1mM) (established previously, data not shown). Where necessary the cells were pre-treated with relevant antagonist/vehicle and incubated for 1 hour. Cells were then incubated for 20-24 hours at 37°C in a humidified atmosphere (95% air, 5% (v/v) CO_2_). Following this the supernatant was removed and stored at -80°C until required for cytokine analysis. All studies were repeated on three separate experimental days. The effect of the two different P2X7 inhibitors on the ATPγS plus LPS enhanced release of inflammasome linked cytokines (and inflammasome independent cytokines i.e. TNFα and IL-6) was determined. Cytokines were measured using ELISA’s from R&D Systems using manufacturer’s instructions.

**Data analysis.**

Data is expressed as mean ± s.e.m of n observations. Statistical significance was determined using either Student’s t-test or One-way ANOVA followed by an appropriate post-hoc test. A P value < 0.05 was taken as significant and all treatments were compared with the appropriate control group.
